# MicroRNA-143 modulates the expression of Natriuretic Peptide Receptor 3 in cardiac cells

**DOI:** 10.1038/s41598-018-25489-3

**Published:** 2018-05-04

**Authors:** Juan Wang, Kai Sing Tong, Lee Lee Wong, Oi-Wah Liew, Divya Raghuram, Arthur Mark Richards, Yei-Tsung Chen

**Affiliations:** 1 Cardiovascular Research Institute, Department of Medicine, Centre for Translational Medicine, Yong Loo Lin School of Medicine, National University of Singapore, Singapore, 117599 Singapore; 2 Cardiac Department, National University Health System, Singapore, 119228 Singapore; 3 Christchurch Heart Institute, University of Otago, Christchurch, 4345 New Zealand

## Abstract

Natriuretic Peptide Receptor 3 (NPR3), the clearance receptor for extracellular bio-active natriuretic peptides (NPs), plays important roles in the homeostasis of body fluid volume and vascular tone. Using luciferase reporter and antagomir-based silencing assays, we demonstrated that the expression of NPR3 could be modulated by microRNA-143 (miR-143-3p), a microRNA species with up-regulated circulating concentrations in clinical heart failure. The regulatory effect of miR-143 on NPR3 expression was further evidenced by the reciprocal relationship between miR-143 and NPR3 levels observed in hypoxia-treated human cardiac cells and in left ventricular tissue from rats undergoing experimental myocardial infarction. Further analysis indicated elevation of miR-143 in response to hypoxic challenge reflects transcriptional activation of the miR-143 host gene (MIR143HG). This was corroborated by demonstration of the induction of host gene promoter activity upon hypoxic challenge. Moreover, miR-143 was shown to enhance its own expression by increasing MIR143HG promoter activity, as well as targeting the expressions of NPPA, NPPC, NR3C2, and CRHR2 in cardiac cells. Taken together, these findings suggest that the elevation of miR-143 upon hypoxic insult may be part of a microRNA-based feed forward loop that results in fine tuning the levels of NPs and neurohormonal receptors in cardiac cell lineages.

## Introduction

The cardiac natriuretic peptides (NPs) together with the sympathetic nervous system and the renin-angiotensin-aldosterone system (SNS-RAAS axis) constitute major components of the neurohormonal signaling network that plays a pivotal role in cardiovascular homeostasis by modulating blood pressure, intravascular volume and vascular tone^1^. The NP family comprises three isoforms, namely, atrial natriuretic peptide (ANP), brain natriuretic peptide (BNP) and C-type natriuretic peptide (CNP)^2^. NPs exert their physiological functions by binding to the cognate G-protein coupled transmembrane receptors, natriuretic peptide receptors 1 and 2 (NPR1 and NPR2), and activating cyclic guanosine monophosphate (cGMP) signaling cascades. All three NPs are rapidly removed from the circulation in part by binding to natriuretic peptide receptor 3 (NPR3) to be subsequently internalized and degraded^3^. Thus, tissue levels of NPR3 plays a critical role in cardiovascular homeostasis by controlling the half-life of bioactive NPs in circulation^2,4^.

Recent genetic research revealed the association of the NPR3 locus with blood pressure in patients^5,6^, suggesting plausible pathological relevance of NPR3 in clinical cardiovascular disease. Ablating NPR3 in mice prolongs the circulating half-life of bioactive ANP with an associated reduction in blood pressure^4^. Intriguingly, other evidence demonstrates the role of the CNP/NPR3 signaling pathway in regulating coronary blood flow suggesting plausible therapeutic value in manipulating NPR3 signaling to ameliorate myocardial ischemia/reperfusion (I/R) injury^7^.

MicroRNAs are short non-coding RNAs (about 21–25 nucleotides) that play important roles in post-transcriptional regulation of almost all biological processes^8,9^. Accumulating evidence has demonstrated the critical roles of microRNAs in various cardiovascular diseases through modulation of underlying signaling networks governing hypertrophy, fibrosis and apoptosis^10,11^. However, it is only in recent years that researchers have taken greater interest in exploring the regulatory effects of microRNAs on the expression of NPs and/or their cognate receptors^12–14^. The genetic variant harboring the rs5068 SNP (A/G) at the 3 prime untranslated region (3′UTR) of NPPA transcripts is associated with blood pressure. Further analyses demonstrated that miR-425 regulates NPPA mRNA level in an allele-specific fashion as evidenced by the disruption of the binding between miR-425 and NPPA 3′UTR for the G allele^12^. Bioinformatics analyses and cellular modeling from the same group identified two additional microRNAs, miR-155 and miR-105, that target the 3′UTR of NPPA. Furthermore, the authors demonstrated that the minor alleles observed in the NPPA 3′UTR are associated with higher plasma ANP levels in patients^14^. In recent published work, our group used multiple platforms to demonstrate the regulatory effects of miR-100 on the expression of NPR3^13^. Notably, miR-100 was found to target NPR3 3′UTR and is differentially upregulated in the plasma of heart failure patients, suggesting a plausible microRNA-based compensatory mechanism in response to cardiac overload. The discovery of microRNA action on NPR3 is particularly interesting, considering that attenuation of the turnover rate of cardio-protective NPs in circulation underpins the development of next generation therapies, such as LCZ696, for heart failure and other cardiovascular diseases^15^. In this report, we explored a cluster of microRNAs predicted to target NPR3-3′UTR and discovered that miR-143, a microRNA previously demonstrated to play pivotal roles in cardiac development and smooth muscle cell differentiation^16^, negatively regulates the expression of NPR3 in cardiac cells derived from human left ventricle. Furthermore, we demonstrated that miR-143 upregulation resulted directly from hypoxia-induced transcriptional activation of its host gene, MIR143HG, as well as by a feed forward effect of miR-143 on its host gene promoter activity.

## Results

### Elevations of miR-143 in HF and experimental platforms

**Figure 1:**
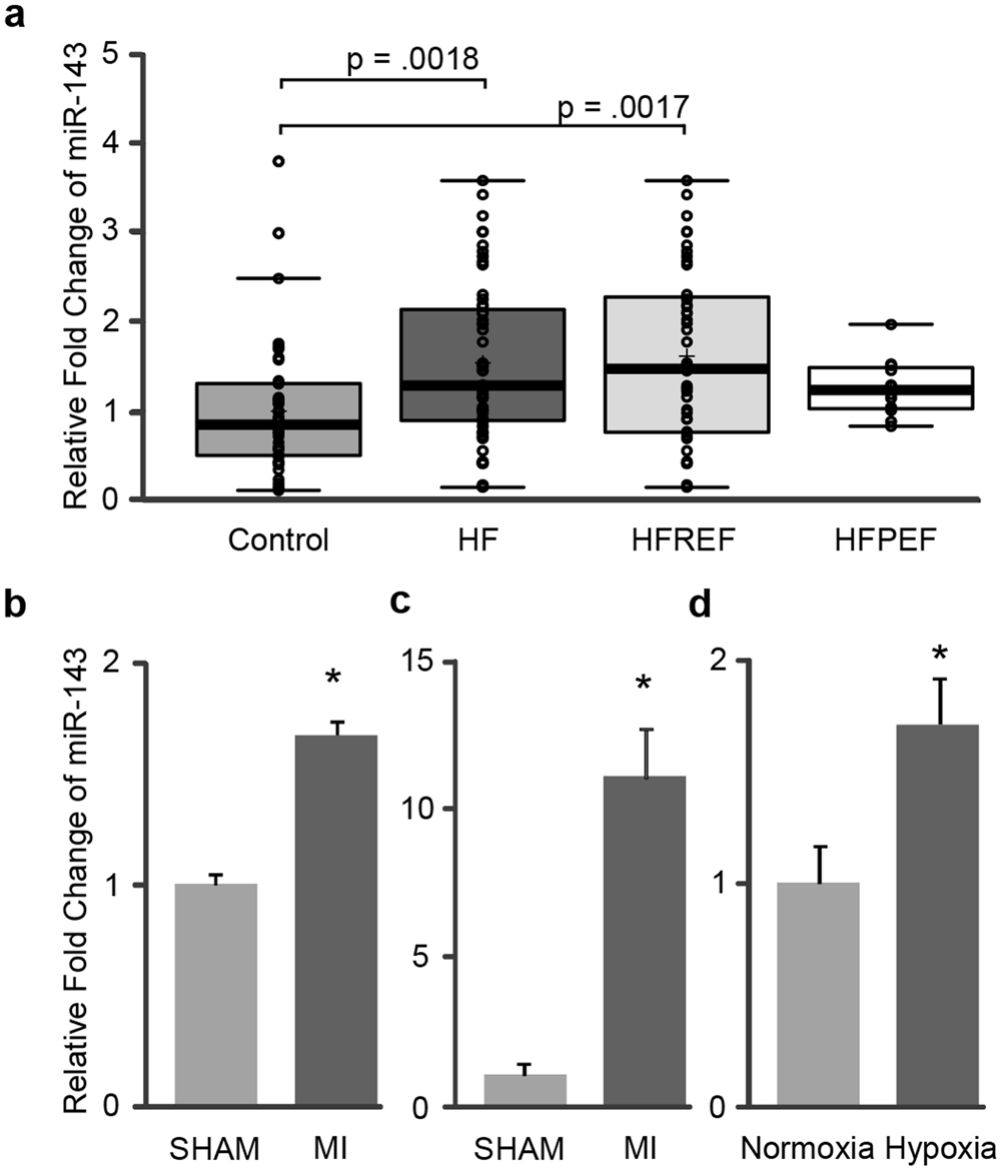
Expression of miR-143 in human HF plasma, animal model and *in vitro* experimental platforms. (**a**) Upregulation of miR-143 in HF plasma compared with non-HF controls. In the Box and Whisker plot, center lines represent the median; lower and upper box limits indicate the 25^th^ and 75^th^ percentiles respectively, crosses represent sample means; data points are plotted as open circles. n = 42, 56, 44, 12 for control, HF, HFREF and HFPEF respectively. Student’s t test, p values as indicated. Relative expression levels of miR-143 in (**b**) rat plasma and (**c**) rat left ventricular tissue at 7 days post-surgery as compared to sham controls (n = 6). (**d**) Expression of miR-143 in HCMa cells after 48 h hypoxic treatment. Data in (**b**,**c** and **d**) are presented as mean ± SD, Student’s t test, *p < 0.05.

Previously, microRNA profiling and microRNA target predicting algorithms were used to identify a subset of upregulated microRNAs targeting the 3′UTR of NPR3 transcripts in cardiac cells subjected to hypoxic treatment^13^. To gain further insights into the possible clinical relevance of hypoxia-induced microRNAs, their circulating levels in heart failure (HF) patients were examined. Among 6 microRNAs investigated, namely miR-143, miR-149, miR-186, miR-222, miR-500, and miR-643, only plasma miR-143 was significantly elevated in 56 HF patients compared with 42 healthy controls (Fig. 1a). The increase in miR-143 in HF was more pronounced in 44 reduced ejection fraction (HFREF) patients (p = 0.0017) than in 12 patients with preserved ejection fraction (HFPEF). To further explore the possible pathological relevance of miR-143 after ischemic insults *in vivo*, a rat model of myocardial infarction (MI) induced by left anterior descending coronary artery ligation was developed to examine subsequent changes in miR-143 gene expression. In agreement with observations in HF patients, miR-143 was found to be significantly up-regulated in MI rat plasma compared with sham control (Fig. 1b). Moreover, the elevation of miR-143 was particularly prominent in the peri-infarct zone of the left ventricle (Fig. 1c), suggesting a cardiac contribution to the increase in circulating miR-143. To confirm the involvement of hypoxia as a trigger for miR-143 upregulation *in vitro*, human left ventricle-derived HCMa cells were cultured in 0.2% oxygen for 48 hours and levels of miR-143 were examined. Compared with normoxic controls, miR-143 levels were elevated in hypoxia-treated HCMa cells (Fig. 1d), as well as in the associated conditioned media (data not shown). Collectively, results from multiple platforms support the postulation that circulating miR-143 could in part originate from hypoxic tissues.

### Regulatory effect of miR-143 on NPR3 expression

**Figure 2:**
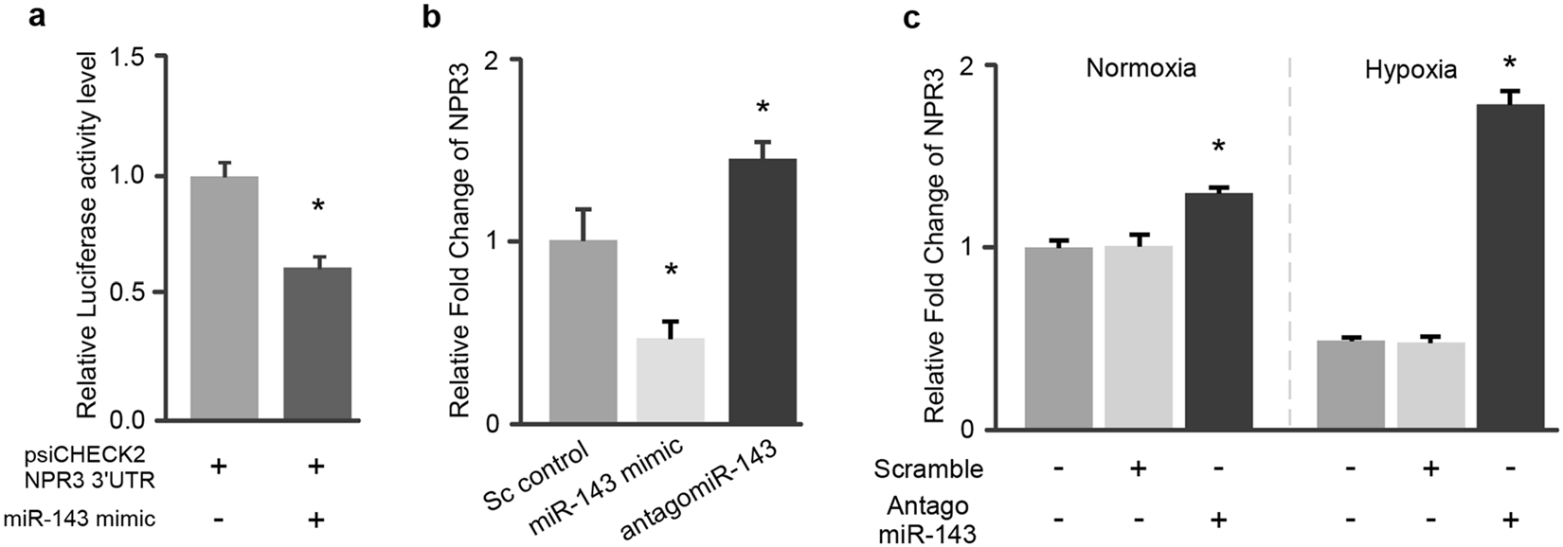
Regulatory effects of miR-143 on the expression of NPR3. (**a**) Luciferase reporter assay to demonstrate the interaction between miR-143 and NPR3 3′UTR. (**b**) Overexpression of miR-143 (miR-143 mimic) suppressed the expression of NPR3 and knockdown of miR-143 (antagomiR-143) increased NPR3 transcripts level in HCMa. (**c**) NPR3 was upregulated in antagomiR-143 treated normoxic cells. Downregulation of NPR3 level in hypoxia-treated HCMa was reversed when co-treated with antagomiR-143. Data are presented as mean ± SD, n = 3, Student’s t test, *p < 0.05.

Results from our previous study have demonstrated that NPR3 levels are downregulated in both hypoxia-treated cardiac cells and infarcted cardiac tissue^13^. *In silico* microRNA target prediction (miRWalk 2.0) identified 5 putative miR-143 binding sites within the 3′UTR of NPR3 (Table 1). To further determine the regulatory effects of miR-143 on NPR3 gene expression, the binding of miR-143 to NPR3 3′UTR was examined using luminescence reporter-based interaction assay. As shown in Fig. 2a, relative luciferase activity in cells co-transfected with psiCHECK2-NPR3 3′UTR and miR-143 mimics was significantly repressed, indicating that the seed region of miR-143 can indeed interact with the NPR3 3′UTR as predicted. Subsequently, HCMa cells were transfected with miR-143 mimics or antagonists and NPR3 transcriptional expression was examined using semi-quantitative PCR. As shown in Fig. 2b, the level of NPR3 transcripts was decreased in cells transfected with miR-143 mimic and significantly elevated in antagomiR-143 treated cells compared with scrambled controls. Under normoxic conditions, we demonstrated significant upregulation of NPR3 in antagomiR-143 treated cells and this effect was exaggerated by hypoxic challenge (Fig. 2c). Collectively, our findings support a role for miR-143 in fine tuning NPR3 transcript levels in cardiac cells under both normoxic and hypoxic conditions.

**Table 1:** Identification of miR-143-3p targeting the NPR3 3′UTR.

miRNA	Gene	RefseqID	miRWalk	Microt4	miRanda	miRMap	PITA	RNA22	RNAhybrid	Targetscan	SUM
hsa-miR-143-3p	NPR3	NM_001204375	1	1	1	1	0	1	1	1	7
		NM_000908	1	0	1	1	1	0	1	1	6
		XM_005248309	1	0	1	1	0	0	1	1	5
		NM_001204376	1	0	1	1	0	0	1	1	5
		XM_005248310	0	0	0	1	0	0	1	0	2

The number of miR-143-3p predicted target sites on NPR3 3′UTR from 8 web-based microRNA target prediction algorithms.

### Hypoxia induces transcriptional activity of miR-143 host gene in HCMa cells

**Figure 3:**
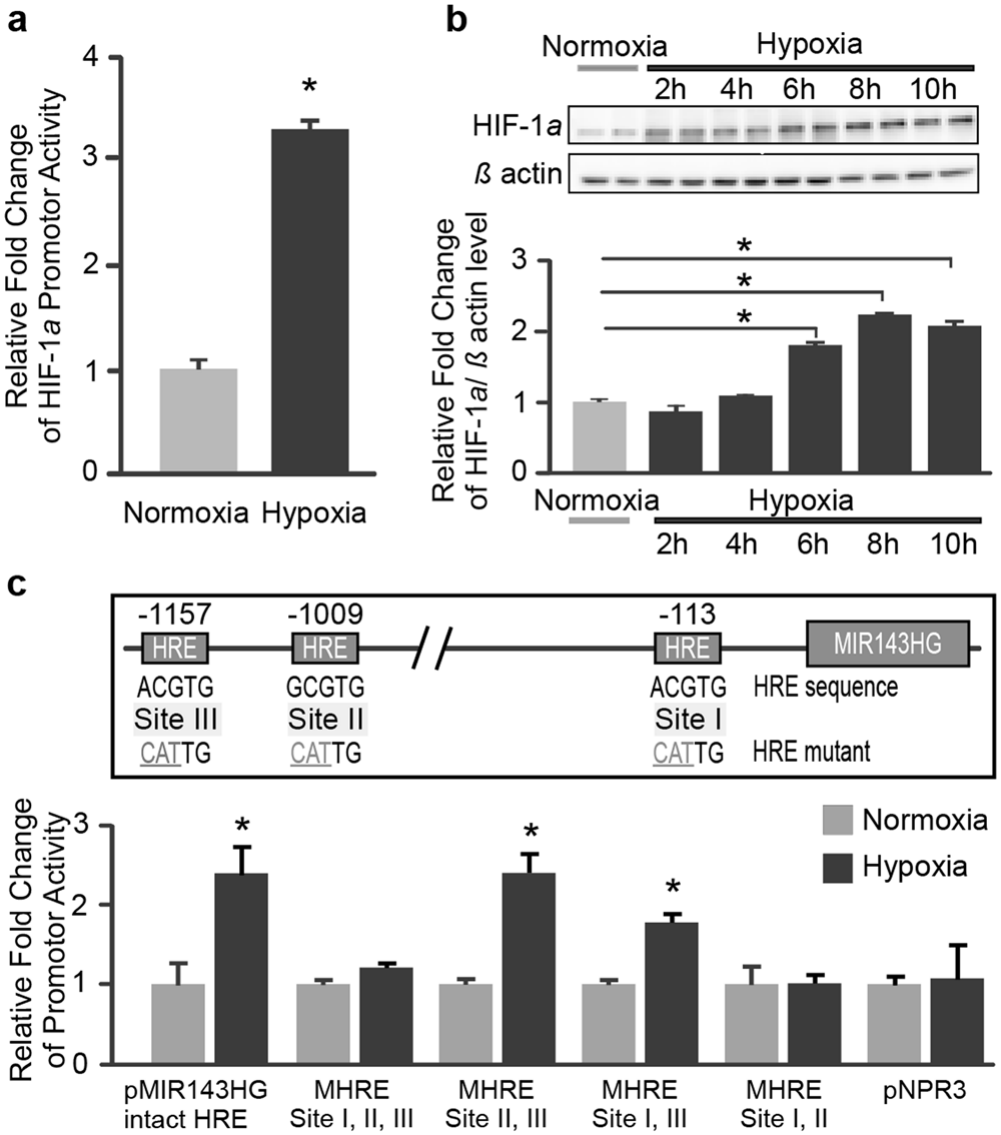
MIR-143HG promoter activity is modulated by hypoxia. (**a**) Hypoxic treatment increases the promoter activity of HIF1a. (**b**) Western blot analysis of HIF-1a protein level in HCMa after hypoxic treatments (**c**) Mutagenesis analyses of MIR143HG promoter demonstrate the essential roles of hypoxia response elements in hypoxia induced transcriptional activation for MIR143HG. The mutated HRE sites are indicated by underlined gray fonts. No change in the NPR3 promoter activity was observed after hypoxia treatment. Data are presented as mean ± SD, n = 3, Student’s t test, *p < 0.05.

Previous studies have shown that miR-143 is encoded by the microRNA-143/145 host non-coding RNA gene (MIR143HG, NR_027180; also known as NCR143/145) and the expression of miR-143 is modulated by transcriptional activation of the MIR143HG promoter^17^. Examination of the promoter region of MIR143HG revealed three hypoxia-inducible factor binding sites (consensus motif A/GCGTG), suggesting miR-143 gene regulation might be controlled via a hypoxia-associated signaling cascade. To elucidate the involvement of hypoxia in the induction of miR-143 in HCMa, promoter activity of the alpha subunit of hypoxia-inducible factor-1 (HIF-1α) was first examined. As shown in Fig. 3a, the promotor activity of HIF-1α gene was significantly enhanced in hypoxia-treated HCMa cells subjected to 0.2% oxygen for 24 hours compared with normoxia control. This result was corroborated by the time-dependent increase in gene expression of HIF-1α transcripts observed in hypoxia-treated HCMa cells (data not shown). Western blot analysis further revealed a gradual increase in HIF-1α protein levels 6 hours after the onset of hypoxia (Fig. 3b). Using promoter activity analysis, we demonstrated that the transcriptional activity of NPR3 remains intact as indicated by a lack of change in the NPR3 promoter activity after hypoxic treatment (Fig. 3c). This result supports possible involvement of microRNA-mediated post-transcriptional regulation of NPR3 gene expression in the cellular response to hypoxic stress.

To further establish the correlation between hypoxic stimulation and transcriptional activation of MIR143HG, an approximately 2-Kb DNA fragment upstream of the start codon of the MIR143HG gene was cloned into the vector pGL4.10 for subsequent luciferase analysis. As shown in Fig. 3c, MIR143HG promoter (pMIR143HG) activity was significantly increased in hypoxia-treated HCMa cells compared with normoxia control. To confirm that transcriptional activation of MIR143HG is mediated by HIF-1a under hypoxic stress, multi-point mutations were introduced into three hypoxia response elements (HRE) located at positions −113 bp (Site I), −1009 bp (Site II) and −1157 bp (Site III)) with respect to the transcription start site on the MIR143HG promoter region (Fig. 3c). The promoter activities of various combinations of these different fragments containing two or three mutated HREs were examined. Luciferase reporter analyses showed that hypoxia induced MIR143HG promoter activities were attenuated or diminished in cells that harbor mutated HRE constructs (Fig. 3c). This finding indicates that HRE motifs in the promotor region are important elements regulating hypoxia-induced transcriptional activity of MIR143HG. Our data indicated two of the three HIF binding sites, namely the −113 and −1009, are required for efficient transcriptional activation of MIR143HG under hypoxic conditions as mutations in both motifs abolished hypoxia-induced MIR143HG promoter activity.

### Feedforward regulation of miR-143 and miR-100 on the MIR143HG promoter activity

**Figure 4:**
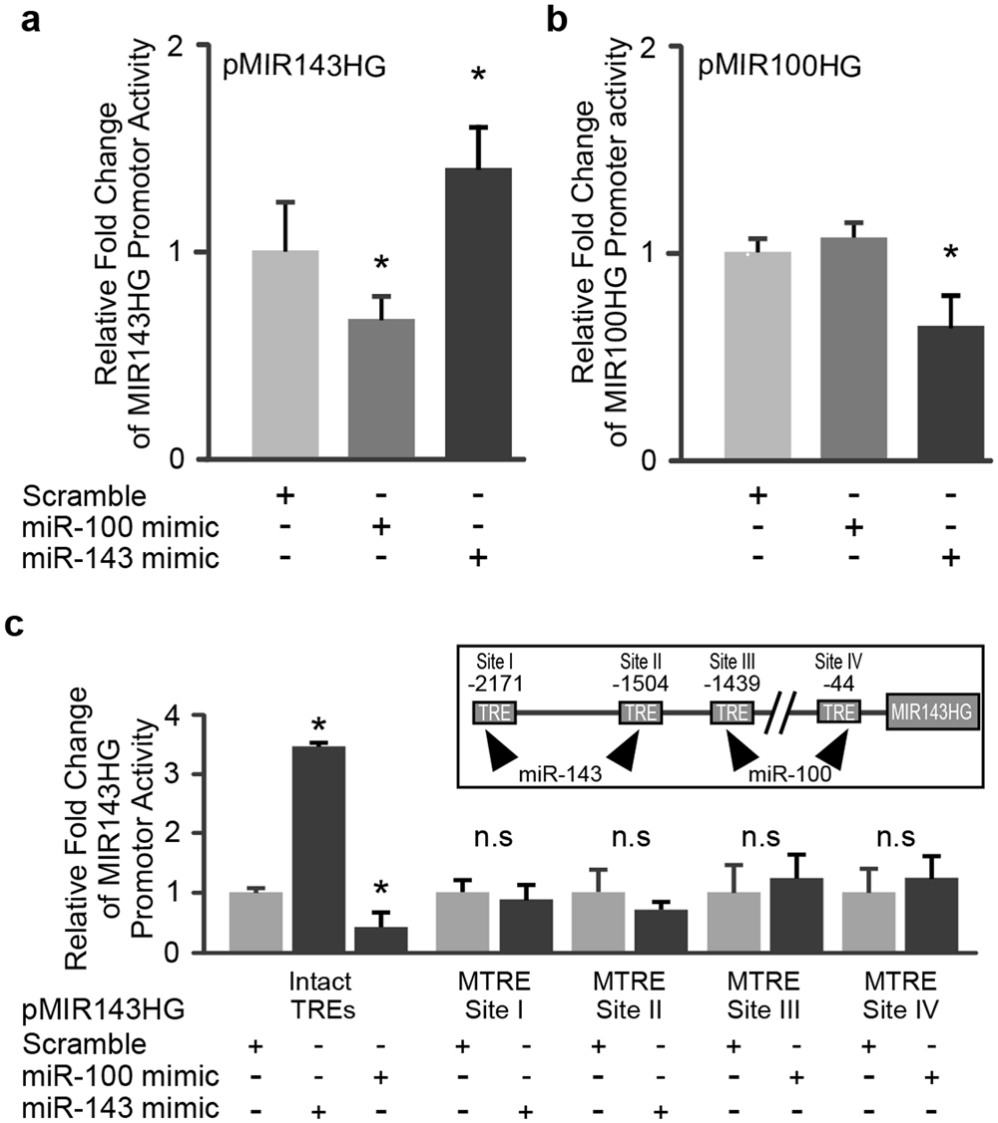
Differential promoter activities of MIR143HG and MIR100HG in the presence of exogenous miR-100 or miR-143 in HCMa cells. (**a**) Promoter activities of MIR143HG were enhanced and inhibited in the presence of exogenous miR-143 and miR-100 respectively. (**b**) Promoter activity of MIR100HG was attenuated in the presence of miR-143 mimics. (**c**) Mutagenesis analyses of MIR143HG promoter showing that miR-143 and miR-100 failed to interact with promoter constructs containing the mutated transcriptional response elements (MTRE) representing the respective microRNA binding sites. Inset in (**c**) depicts the relative locations of the TREs tested in this study. Data are presented as mean ± SD, n = 3, Student’s t test, *p < 0.05. n.s, non-significance, p > 0.05.

Sequence analysis of the promoter region of MIR143HG revealed the presence of several specific sequence motifs (~6–7 nucleotides) that are complementary to mature miR-143 and miR-100. In the 2-Kb MIR143HG promoter region, 7 and 4 putative binding sites were identified for miR-143 and miR-100, respectively (Tables 2 and 3). Analysis using the PROMO software (http://alggen.lsi.upc.es) further revealed that these putative microRNA binding sites overlap with numerous transcription factor binding sites, suggesting that the binding of these two microRNAs in the MIR143HG promoter region may interfere with its transcriptional activity. To examine the possible microRNA-directed transcriptional regulation of MIR143HG, miR-143 and miR-100 mimics were co-transfected with the pMIR143HG-containing vector and the relative strength of promoter activities in driving luciferase reporter expression in transfected cells was determined. Intriguingly, the promoter activity of MIR143HG was enhanced in the presence of exogenous miR-143, but was suppressed by the exogenous miR100 compared to scrambled controls (Fig. 4a). The negative modulation of the transcriptional activity of miR-100 host gene, MIR100HG, was also observed when treated with exogenous miR-143 (Fig. 4b). This finding was corroborated by the observed upregulation of miR-100 in multiple cardiovascular cell lineages after knock down of the endogenous miR-143 levels using antagomiR-143 (Supplemental data 1). While miR-100 and miR-143 target NPR3 to downregulate its expression, both microRNAs were also observed to regulate MIR143HG promoter activity suggesting possible cross talk between the two NPR3-targeting microRNAs.

**Table 2:** Position and nucleotide sequences of overlapping regions between putative miR-143 target sites and transcriptional motifs on the MIR143HG promoter region.

Location		Sequence alignment	Transcriptional factors
−1038	pMIR143HG miR-143	5′-**GAGCTA**GCCA**GC**AAGAAACT**A**-3′ 3′-**CUCGAU**GUCA**CG**AAGUAGAG**U**-5′	ENKTF-1
−1504	pMIR143HG miR-143	5′-C**AG**GG**ACAGTG**TC**T**G**AT**TCAG-3′ 3′-C**UC**GA**UGUCAC**GA**A**G**UA**GAGU-5′	TFII-I, AR
−1891	pMIR143HG miR-143	5′-AG**G**AG**ACAGTG**A**T**CTTCACAG-3′ 3′-CU**C**GA**UGUCAC**G**A**AGUAGAGU-5′	—
−1900	pMIR143HG miR-143	5′-**G**T**GCTACAG**A**G**GAGACAG**T**G**A**-3′ 3′-**C**U**CGAUGUC**A**C**GAAGUAG**A**G**U**-5′	GR-α
−2064	pMIR143HG miR-143	5′ -ACATGAAC**G**GAA**TTCATCT**TC-3′ 3′-CUCGAUGU**C**ACG**AAGUAGA**GU-5′	XBP-1, NFI/CTF
−2106	pMIR143HG miR-143	5′-CTT**C**CCT**A**ACC**C**AC**CATCTC**T-3′ 3′-CUC**G**AUG**U**CAC**G**AA**GUAGAG**U-5′	YY1, coup-TF1
−2171	pMIR143HG miR-143	5′-TGT**C**AGTG**GTGCTT**TGGAGT**A**- 3′ 3′-CUC**G**AUGU**CACGAA**GUAGAG**U**-5′	NFI/CTF, SRY, GR, TCF and LEF-1

The promoter position of the nucleotide with respect to the transcription start site (defined as position 0) that is aligned to the 3′ end of each putative miR-143 binding site in MIR143HG promoter region is indicated under the “Location” column. Underlined bolded letters indicate the complementary sequences for putative binding between the microRNA seed region and transcription factor motifs on the MIR143HG promoter.

**Table 3:** Position and nucleotide sequences of overlapping regions between putative miR-100 target sites and transcriptional motifs on the MIR143HG promoter region.

Location		Sequence alignment	Transcriptional factors
−44	pMIR143HG miR-100	5′-C**C**CGCCTCG**C**CCCAA**TACGGG**GC-3′ 3′-**G**UGUUCAA**G**CCUAG**AUGCCC**AA-5′	GR-β, Pax-5, P53 and NFI/CTF
−639	pMIR143HG miR-100	5′-GCTGG**GT**CA**GGATCT**T**C**CCTG**T**-3′ 3′-GUGUU**CA**AG**CCUAGA**U**G**CCCA**A**-5′	TFII-I, STAT4, NF-κB, RelA and RAR-α
−1439	pMIR143HG miR-100	5′-**CACAAG**GC**C**CCG**T**G**T**G**C**T**G**C**T**G-3′ 3′-**GUGUUC**AA**G**CCU**A**G**A**U**G**C**C**C**A**A-5′	GR-α, AP-2αA, Pax-5 and P53
−2112	pMIR143HG miR-100	5′-**CACAAG**CCTTCCCTA**AC**CCACC-3′ 3′-**GUGUUC**AAGCCUAGA**UG**CCCAA-5′	C/EBPβ

The promoter position of the nucleotide with respect to the transcription start site (defined as position 0) that is aligned to the 3′ end of each putative miR-100 binding site in MIR143HG promoter region is indicated under the “Location” column. Underlined bolded letters indicate the complementary sequences for putative binding between the microRNA seed region and transcription factor motifs on the MIR143HG promoter.

To further examine the interaction of miR-100 and miR-143 with predicted target sites on the promoter region of MIR143HG, mutations were introduced into selected transcriptional response elements that contain over 90% complementary base pair matching with the mature seed region of micro-143 and miR-100, namely −1504 bp and −2171 bp (miR-143 target sites), as well as −44 bp, and −1439 bp (miR-100 target sites) with respect to the transcription start site on the MIR143HG promoter region. Luciferase reporter analyses showed that miR-100 and miR-143 fail to modulate the luciferase activities in cardiac cells that harbor the corresponding mutated MIR143HG promoter constructs (Fig. 4a). This finding indicates that complementary base pairing of miR-100 and miR-143 with predicted motifs in the promotor region might play role(s) in microRNA-directed transcriptional modulation of the expression of MIR143HG.

### MiR-143 targets other neurohormones and associated receptors

**Figure 5:**
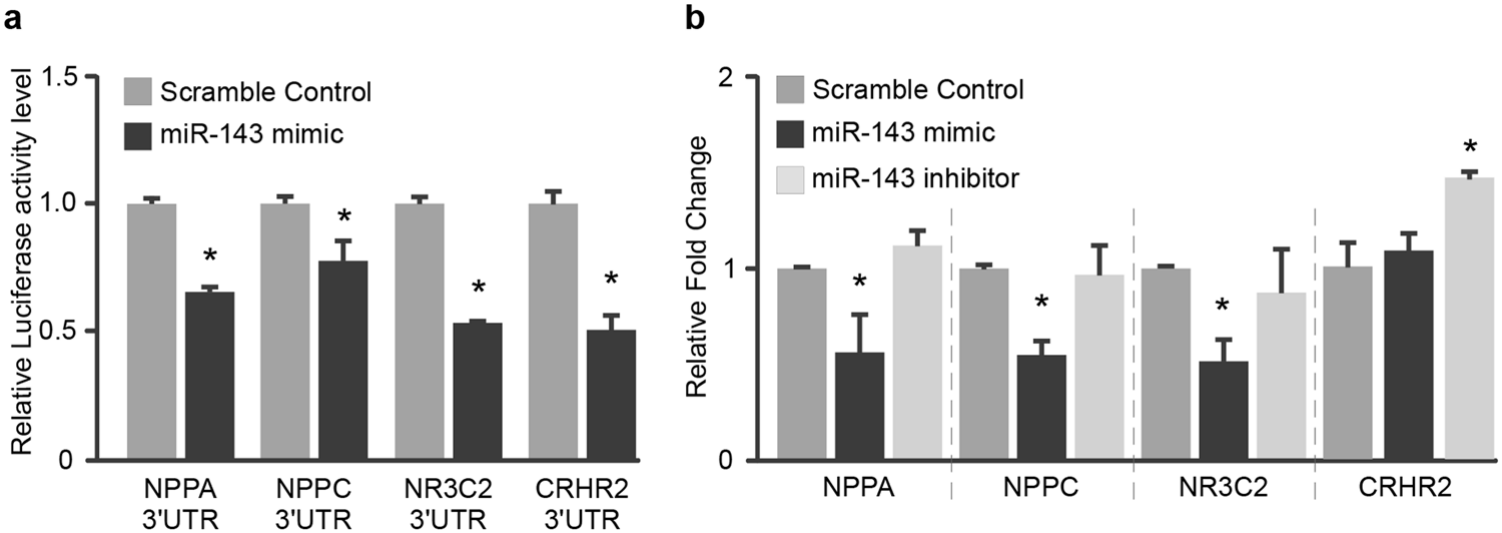
Regulatory effects of miR-143 on the expression of NPPA, NPPC, NR3C2 and CRHR2. (**a**) Luciferase reporter assay to demonstrate the interactions between miR-143 and the 3′UTRs of NPPA, NPPC, NR3C2 and CRHR2. (**b**) Overexpression of miR-143 (miR-143 mimic) suppressed expression of NPPA, NPPC, and NR3C2 in HCMa cells. However, knockdown of NPR3 level with antagomiR-143 did not lead to enhancement of NPPA, NPPC, and NR3C2 levels in HCMa cells. Data are presented as mean ± SD, n = 3, Student’s t test, *p < 0.05.

We recently reported considerable discrepancy between the putative microRNA-target pairs identified by *in silico* microRNA target prediction algorithms and those from *in vitro* functional assessment by microRNA/3′UTR luciferase assays^18^. Careful validation by cell-based reporter assays revealed that both *in silico*-based and data-driven prediction algorithms returned a high proportion of false negative and false positive results. Thus, to explore the posited regulatory effects of miR-143 on other neurohormones and associated enzymes/receptors, the interactions of miR-143 with a panel of neurohormonal 3′UTR constructs were first evaluated using luciferase reporter assays. Intriguingly, in addition to NPR3, four additional neurohormonal-related genes were found to interact with miR-143, including natriuretic peptide A (NPPA), natriuretic peptide C (NPPC), and nuclear receptor subfamily 3, group C, member 2 (NR3C2), and corticotropin releasing hormone receptor 2 (CRHR2) (Fig. 5a). Gain and loss of function assays were then performed in HCMa cells to examine the regulatory effects of miR-143 on the expression of these putative target genes. As shown in Fig. 5b, overexpression of miR-143 repressed NPPA, NPPC and NR3C2 expression in HCMa cells, while knockdown of miR-143 upregulated expression of CRHR2. Corroborating these findings, differential expression of these genes of interest were also observed in the other cardiac cell lines after manipulation of endogenous miR-143 levels using analogs or antagomirs (Supplemental data 2). These results indicate miR-143 may play multiple roles in circulatory homeostasis by modulating the expression of a range of relevant neurohormones and/or their associated receptors.

## Discussion

Suppression of NPs turnover through inhibition of neprilysin activity has been shown to ameliorate symptoms of heart failure, catapulting this protease as a therapeutic target for developing next generation medicines^19,20^. Similar to neprilysin, NPR3 is a clearance receptor that mediates internalization and degradation of bioactive NPs in circulation and therefore appropriate regulation of cellular levels of NPR3 is important for maintaining cardiovascular homeostasis^2,4^. It is known that NPR3 is ubiquitously expressed in mammals and kidney, heart muscle, thyroid, and adipose tissues in particular are known to have the highest mRNA expression levels. Hence, studies clarifying the mechanistic actions of miR-143 in modulating NPR3 gene expression may provide an alternative or supplementary approach in developing future HF therapeutics by prolong the half-life of NPs.

Findings from previous studies using various experimental platforms have suggested important roles for miR-143 in the cardiovascular system. In zebrafish heart, miR-143 has been shown to be essential for cardiac chamber morphogenesis by targeting adducin 3^21^. The role of miR-143 in mouse vascular smooth muscle cells (VSMCs) differentiation was demonstrated by targeting Ets-like gene 1 (ELK1), a transcriptional coactivator that is crucial for regulation of the VSMC phenotype^16^. Recently, a link between miR-143 and cardiovascular disease was suggested by observations of elevated miR-143 levels in patients with pulmonary artery hypertension (PAH), as well as in animal models of PAH^17^. In that study, miR-143 was found to be highly enriched in pulmonary artery smooth muscle cell (PASMC)-derived exosomes suggesting a role as a biological messenger mediating crosstalk between PASMCs and endothelial cells. In experimental PAH models, the authors further demonstrated that downregulation of miR-143 attenuates pulmonary vascular remodeling and the development of pulmonary arterial hypertension^17^. A recent genetic study on ischemic stroke (IS) discovered the association of polymorphisms within the promoter region of the MIR143HG with the risk of IS and essential hypertension^22,23^, providing additional evidence for the functional relevance of miR-143 in cardiovascular diseases. Importantly, results from our study demonstrate that miR-143 may modulate multiple molecules involved in cardiovascular neurohormonal signaling in cardiac lineages, suggesting miR-143 modulates expression of several genes relevant to cardiovascular biology and function.

Our finding of the inhibitory effect of miR-143 on NPR3 expression in cardiac cells is particularly intriguing as knockdown of NPR3 in rats, using RNA interference, elevates circulating ANP levels and ameliorates isoproterenol-induced cardiac hypertrophy^24^. Hence targeting of NPR3 with miR-143 may increase ANP bioactivity to augment the cardioprotective effects of NP signaling. Our current data also suggest possible beneficial physiological consequences of microRNA mediated downregulation of NPR3 in the setting of hypoxic insults to cardiac tissues. Western blotting analyses revealed distinct NPR3 expression patterns across three cardiac cell lines (derived from different donors) when subjected to normoxic or hypoxic conditions in the presence of exogenous miR-143 mimic or antagomir (Supplemental data 3). Consistent with our previous observations, miR-143 was upregulated in all three cardiac cell lines examined after hypoxic treatment (Fig. 1d and Supplemental data 1). With hypoxic challenge, downregulation of NPR3 protein level was observed in HCMa and ATCC cardiac cells but not in PromoCell cardiac cells. Intriguingly, only HCMa and PromoCell cardiac cells responded to miR-143 antagomir treatment with increased NPR3 protein levels under normoxic condition. None of the tested cardiac cells responded to miR-143 mimic treatments as judged from the Western blotting analyses. These results are not totally unexpected as it is known that global gene transcript patterns can vary between different cell types, in part reflecting intrinsic differences in intracellular regulation of signaling networks governing synthesis, editing and degradation of transcripts and protein molecules. Hence, the regulatory effect of miR-143 on NPR3 transcript levels might be only one of varied post-transcriptional modification mechanisms present in cells. As a result, the effects of mimic or antagomir treatments on NPR3 protein levels may be subtle and/or masked by other cellular processes governing translational efficiency and protein turnover rates.

However, it is noteworthy that in addition to being a clearance pathway for NPs in vascular cells CNP binding to NPR3 is reported to exert cardioprotective effects by coupling with inhibitory G protein and activating phospholipase C signaling^7,25^. In H9C2 cells, NPR3 plays a role in apoptosis by mediating the activity of cAMP response element binding protein (CREB) and downstream signaling involving breast cancer type 1 susceptibility protein (BRCA1) and tumor necrosis factor α (TNF α)^26^. The complexity of the NPR3-directed downstream signaling cascade is further demonstrated by the phenotypic manifestation of skeletal deformities in NPR3-ablated mice^4^, and *in vitro* data suggesting putative roles in proliferation and migration in various of cellular lineages^27–29^. As NPR3 is ubiquitously expressed in most cell types, it would be interesting in future work to further delineate the effects of microRNA-mediated post-transcriptional NPR3 modification in different cell lineages in the context of varied disease states.

Examination of the promoter region of MIR143HG revealed binding sites for several transcriptional factors including serum response factor, myocardin, and NK2 transcription factor related locus 5 (NKx2.5), Jag-1/Notch and Ras responsive element binding protein (RREB1), and these interactions have been demonstrated to play important roles in smooth muscle cell differentiation^16,30^. Although two HREs were previous identified in the promoter region of MIR143HG, the functional consequence of induction of miR-143 in hypoxic insults is unknown. Using site-directed mutagenesis of MIR143HG promoter HREs, we have shown for the first time that miR-143 gene regulation may be controlled via a hypoxia-associated signaling cascade. This finding provides the molecular basis for the association of miR-143 with ischemic-related cardiovascular diseases and indicates that miR-143 functions as a modulator of the cardiac cellular response to oxygen deprivation.

Recent analyses by microRNA target prediction software have shown that the seed regions of microRNAs may bind not only to the target regions in the 3′UTR of genes, but also guide the RISC to the other genomic regions including promoter regions of genes to elicit other regulatory functions. Two groups have reported that microRNAs can regulate gene expression by microRNA-directed transcriptional activation termed microRNA activation (RNAa)^31,32^. In the current study, the effects of miR-143 and miR-100 on MIR143HG and MIR100HG promoter activities suggest possible cross talk between the two microRNAs. Conceivably, subtle changes in the relative abundance of these two microRNAs may amplify enhancing or attenuating effects on promoter activity. At present, the detailed mechanisms by which microRNAs direct transcriptional regulation of promoter activity remains to be clarified. Nevertheless, the changes in MIR143HG promoter activities after treatment with miR-100 and miR-143 mimics reflect the complexity of microRNA-directed regulatory mechanisms in gene expression. Hence, overlap of predicted microRNA binding sites with numerous transcriptional factor binding sites raises the possibility that microRNA could directly interfere with promoter activity by competing with transcriptional factor binding motifs. This hypothesis is supported by two lines of evidence, first, the observation of diminished regulatory effect of miR-100 and miR-143 on luciferase activity in cells that harbor mutations on the MIR143HG promoter. Second, the upregulated miR-100 levels observed in multiple cardiovascular cells after knock down of miR-143 expression under both normoxic and hypoxic conditions (Supplemental data 1).

**Figure 6:**
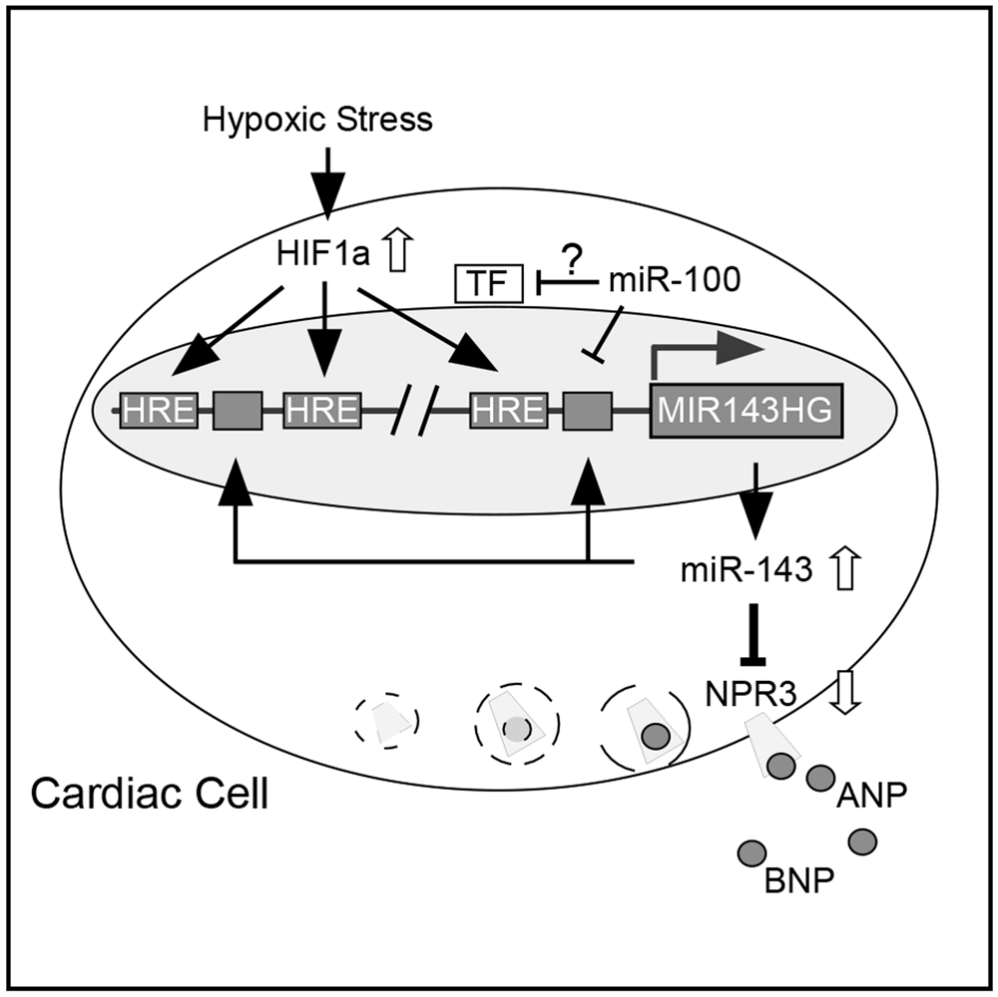
Schematic diagram of hypoxia-induced transcriptional activation of MIR143HG and the proposed microRNA directed transcriptional modification by miR-143 and miR100 in cardiac cells.

Given the importance of NPs in pressure-volume homeostasis, mechanisms for NP synthesis and clearance are both critical for optimizing levels of vasoactive peptide hormones. Luciferase reporter-based assays together with gain- and loss- of function tests provided strong evidence for the regulatory effects of miR-143 on the expression of NPR3 in cardiac cells. Moreover, identification of HRE motifs in the promoter of MIR-143HG confirmed the transcriptional activation of MIR-143HG under hypoxic conditions (Fig. 6). As NPs, particularly ANP and BNP, are known to exert cardio-protective effects countering the deleterious pathological consequences arising from activation of the RAAS and sympathetic nervous systems in cardiovascular disease states, our results suggest that induction of miR-143 may potentially be exploited for therapeutic manipulation of the bioactivity of circulating and /or tissue based cardioprotective NPs.

## Methods

### Cell culture

Hela cells were cultured in Dulbecco’s Modified Eagle Medium/Nutrient Mixture F-12 (DMEM/F-12, Thermo Fisher Scientific) containing 10% Fetal Bovine Serum (FBS, Sigma). Human left ventricle-derived cardiac cells (HCMa) were bought from ScienCell Research Laboratories (Carlsbad, CA, USA) and cultured in cardiac myocyte medium (CMM, ScienCell Research Laboratories) according to manufacturer’s protocol. Both cells were cultured at 37 °C in an incubator with 5% CO2 and proliferated according to protocols for downstream experiments.

### Hypoxia induced experiments

HCMa cells were cultured in a 37 °C incubator controlled with 0.2% O2, 5% CO2 and 94.8% N2 (BioSpheric, Lacona, NY, USA) at different time points.

### Heart failure clinical cohort and rat model of myocardial infarction

These experiments were performed as described^13^. The clinical cohort study protocol was approved by the National Health Group Domain Specific Review Board (NHG DSRB Reference Code: 2010/00114) and Institutional Review Board of National University of Singapore (NUSIRB Reference Code: 04-140) respectively. All patients and control subjects provided written informed consent. The experimental protocols for rat model of myocardial infarction was approved by the Institutional Animal Care and Use Committee (IACUC) of the National University of Singapore and carried out in accordance with established institutional guiding principles for animal research.

### RNA extraction and real-time PCR

Total RNA was extracted with TRIzol® Reagent (Life technologies) following the manufacturer’s protocol. High-Capacity cDNA Reverse Transcription Kit (Applied Biosystems) was used to synthesize the cDNA from RNA. NPR3 and GUSB (reference gene) mRNA levels were measured with iTaq Universal SYBR Green Supermix (Bio-rad, USA) in a QuantStudio™ 7 Flex Real-Time PCR System (Life Technologies) according to the manufacturer’s protocol. The sequence information of the real-time PCR primers are shown below:

- NPR3 forward primer: 5′-AGTTGAGAAACAAGGGCTCAA-3′;
- NPR3 reverse primer: 5′-AGCCAAGACGTAGAGGAGGA-3′;
- GUSB forward primer: 5′-CTCATTTGGAATTTTGCCGATT-3′;
- GUSB reverse primer: 5′-CCGAGTGAAGATCCCCTTTTTA-3′;

For quantification of microRNA expression levels, TaqMan microRNA reverse transcription and real-time assay kits (Applied Biosystems) were used according to manufacturer’s protocol. U6 small nuclear RNA (U6 snRNA) was used as reference microRNA.

### Luciferase Reporter Assay

The 1-kb 3′UTR sequence of NPR3 was cloned into psiCHECK-2 vectors (Promega)^13^. One hundred and fifty ng psiCHECK-2-NPR3-3′UTR plasmid and 10 pmol mature microRNA-143 mimic (Ambion™) were co-transfected into HeLa cells with Lipofectamine 2000 (Life Technologies) in a 24-well plate. Cells lysates were analyzed with Dual-Luciferase® Reporter Assay System (Promega) according to manufacturer’s protocol at 48 h after transfection.

Promoter fragments comprising approximately 2-Kb of DNA sequences immediately upstream from the transcription start sites of NPR3, MIR143HG, and MIR100HG were cloned into pGL4.10 (Promega) vectors respectively for promoter activity assay. The primers for the promoter fragments are indicated below:

- NPR3 promoter forward primer: 5′-CTAGCTAGCCTGTGCTGCCATAAACGCCT-3′;
- NPR3 promoter reverse primer: 5′-ATCGGATATCCGTGCCGCAAGAAAGAGCTT-3′;
- MIR143HG promoter forward primer: 5′-ATATGGTACCCTAGAGTCTGTCAGTGGTGC-3′;
- MIR143HG promoter reverse primer: 5′-ATATAAGCTTCAGAGGCCACTCTGCCTTTT-3′
- MIR100HG promoter forward primer: 5′- ACCGCTCGAGTGTAGAAGTAATTCTGGAT-3′;
- MIR100HG promoter reverse primer: 5′-GAATAAGCTTTGCGCTTCCAAAATTCCGCA-3′.

The NPR3 and MIR143 promoter fragments were inserted upstream of the luciferase reporter coding sequence which is flanked downstream by the NPR 3′ UTR. The primers for the NPR 3′UTR are indicated below:

- NPR3 3′UTR forward primer: 5′-GTCACTCGAGCAGTAGCTTAAAGGGAAGCCC-3′;
- NPR3 3′UTR reverse primer: 5′-GAATGCGGCCGCATGTGCTTGCTGTCAAGTAG-3′;

Five hundred ng pGL4.1 promoter plasmid and 5 ng pGL4.73 positive control plasmid were co-transfected into HCMa cells with Lipofectamine 3000 (Life Technologies) in a 24-well plate. Hypoxia or mimics were applied to the transfected cells as required for the various experiments. Cells lysates were analyzed with Dual-Luciferase® Reporter Assay System (Promega) according to manufacturer’s protocol at 6 h/24 h after transfection.

### HIF-1A promoter activity analysis

Cignal Finder Stress & Toxicity 10-Pathway Reporter Array (CCA-007L, QIGEN) was used according to the manufacturer’s protocol to test HIF-1A promoter activity in HCMa cells subjected to 24-h hypoxia treatment. HCMa cells were seeded in a 24-well plate (20,000 cells per well), and transfected with hypoxia reporter, HIF-1A using Lipofectamine 3000 (Life Technologies). After 24-h culture under normoxic or hypoxic conditions, cells were lysed and HIF-1A driven reporter gene expression analysed using Dual-Luciferase® Reporter Assay System (Promega).

### Western blot analysis

Western blotting was performed as described previously. Briefly, HCMa cells were homogenized in cold RIPA lysis buffer (Sigma) containing freshly added 1% protease inhibitor cocktail (Sigma). BCA protein assay (Pierce) was used for protein quantification according to manufacturer’s protocol. Proteins (25 ug/lane) were separated by 10% SDS-PAGE gel and transferred to PVDF membranes. The membranes were blocked in StartingBlock™ T20 (TBS) Blocking Buffer (Thermo Fisher Scientific) for 20 min. Primary antibodies, HIF-1A (sc-10790, 1:200; Santa Cruz) and β-actin (sc-47778, 1:10000; Santa Cruz) were used and the blots were incubated overnight at 4 °C. The membranes were then incubated in corresponding secondary antibodies, Goat anti-Mouse-IgG conjugated to HRP (Sigma) and Goat anti-rabbit-IgG conjugated to HRP (Sigma) in 1:2000 dilution. The relative protein expression levels were normalized against β-actin. Image J was used for band intensity quantification.

### Site-directed mutagenesis

Promoter plasmid pGL4.1-MIR143HG was used as template for site-directed mutagenesis. GeneArt® Site-Directed Mutagenesis PLUS System (A14604, Thermo Fisher Scientific) was applied according to manufacturer’s protocol to obtain the mutated versions of the HRE I, II and III sites on the MIR143HG promoter fragment. All the primers sequences are listed below:

-113M-FP: 5′-CAAGGCAAGGTAGTCCATTGGGGGGTGCCTGGG-3′;
-113M-RP: 5′-CCCAGGCACCCCCCAATGGACTACCTTGCCTTG-3′;
-1009M-FP: 5′-GTAAGATAGGCACATCATTGGCGAGTCCGAAGC-3′;
-1009M-RP: 5′-GCTTCGGACTCGCCAATGATGTGCCTATCTTAC-3′;
-1157M-FP: 5′-GCCGAGGCCTGGTTCCATTGATCCCTTGATTTG-3′;
-1157M-RP: 5′-CAAATCAAGGGATCAATGGAACCAGGCCTCGGC-3′.

Mutations are underlined. All the mutations were confirmed by DNA sequencing.

### Statistical analysis

Student’s t-test was used to compare the relative expression of NPR3, HIF-1A and miR-143 at different time points of hypoxia treatment, MI vs sham as well as between clinical groups of heart failure patients and controls. The significance level was set at p < 0.05. All *in vitro* assays were carried out with at least 3 independent experiments and data was expressed as mean ± SD as indicated. For clinical samples, data was represented as mean ± SD.

## Electronic supplementary material

Supplementary Information

## References

[1] Maack, T. The broad homeostatic role of natriuretic peptides. *Arq Bras Endocrinol Metabol* **50**, 198–207, S0004-27302006000200006 (2006). 10.1590/s0004-27302006000200006 16767286

[2] Pandey KN (2005). Biology of natriuretic peptides and their receptors. Peptides.

[3] Maack T (1987). Physiological role of silent receptors of atrial natriuretic factor. Science.

[4] Matsukawa N (1999). The natriuretic peptide clearance receptor locally modulates the physiological effects of the natriuretic peptide system. Proc Natl Acad Sci USA.

[5] Zhu X (2011). Combined admixture mapping and association analysis identifies a novel blood pressure genetic locus on 5p13: contributions from the CARe consortium. Hum Mol Genet.

[6] International Consortium for Blood Pressure Genome-Wide Association, S. *et al*. Genetic variants in novel pathways influence blood pressure and cardiovascular disease risk. *Nature* **478**, 103–109, 10.1038/nature10405 (2011). 10.1038/nature10405 PMC3340926 21909115

[7] Hobbs A, Foster P, Prescott C, Scotland R, Ahluwalia A (2004). Natriuretic peptide receptor-C regulates coronary blood flow and prevents myocardial ischemia/reperfusion injury: novel cardioprotective role for endothelium-derived C-type natriuretic peptide. Circulation.

[8] Bartel DP (2009). MicroRNAs: target recognition and regulatory functions. Cell.

[9] Ha M, Kim VN (2014). Regulation of microRNA biogenesis. Nat Rev Mol Cell Biol.

[10] Wang, J., Liew, O. W., Richards, A. M. & Chen, Y. T. Overview of MicroRNAs in Cardiac Hypertrophy, Fibrosis, and Apoptosis. *Int J Mol Sci* **17**, 10.3390/ijms17050749 (2016). 10.3390/ijms17050749 PMC4881570 27213331

[11] Quiat D, Olson EN (2013). MicroRNAs in cardiovascular disease: from pathogenesis to prevention and treatment. J Clin Invest.

[12] Arora P (2013). Atrial natriuretic peptide is negatively regulated by microRNA-425. J Clin Invest.

[13] Wong LL (2015). Natriuretic peptide receptor 3 (NPR3) is regulated by microRNA-100. J Mol Cell Cardiol.

[14] Wu C (2016). Novel MicroRNA Regulators of Atrial Natriuretic Peptide Production. Mol Cell Biol.

[15] Hubers SA, Brown NJ (2016). Combined Angiotensin Receptor Antagonism and Neprilysin Inhibition. Circulation.

[16] Cordes KR (2009). miR-145 and miR-143 regulate smooth muscle cell fate and plasticity. Nature.

[17] Deng L (2015). MicroRNA-143 Activation Regulates Smooth Muscle and Endothelial Cell Crosstalk in Pulmonary Arterial Hypertension. Circ Res.

[18] Chen, Y. T. *et al*. The association of heart failure-related microRNAs with neurohormonal signaling. *Biochim Biophys Acta*, 10.1016/j.bbadis.2016.12.019 (2017). 10.1016/j.bbadis.2016.12.019 28065846

[19] Suematsu Y (2016). LCZ696, an angiotensin receptor-neprilysin inhibitor, improves cardiac function with the attenuation of fibrosis in heart failure with reduced ejection fraction in streptozotocin-induced diabetic mice. European journal of heart failure.

[20] Bayes-Genis A, Barallat J, Richards AM (2016). A Test in Context: Neprilysin: Function, Inhibition, and Biomarker. Journal of the American College of Cardiology.

[21] Deacon DC (2010). The miR-143-adducin3 pathway is essential for cardiac chamber morphogenesis. Development.

[22] Wei YS, Xiang Y, Liao PH, Wang JL, Peng YF (2016). An rs4705342 T > C polymorphism in the promoter of miR-143/145 is associated with a decreased risk of ischemic stroke. Sci Rep.

[23] Fu X, Guo L, Jiang ZM, Zhao LS, Xu AG (2014). An miR-143 promoter variant associated with essential hypertension. Int J Clin Exp Med.

[24] Venkatesan B, Tumala A, Subramanian V, Vellaichamy E (2016). Transient silencing of Npr3 gene expression improved the circulatory levels of atrial natriuretic peptides and attenuated beta-adrenoceptor activation- induced cardiac hypertrophic growth in experimental rats. Eur J Pharmacol.

[25] Rose RA, Giles WR (2008). Natriuretic peptide C receptor signalling in the heart and vasculature. J Physiol.

[26] Lin D, Chai Y, Izadpanah R, Braun SE, Alt E (2016). NPR3 protects cardiomyocytes from apoptosis through inhibition of cytosolic BRCA1 and TNF-alpha. Cell Cycle.

[27] Prins BA (1996). Atrial natriuretic peptide inhibits mitogen-activated protein kinase through the clearance receptor. Potential role in the inhibition of astrocyte proliferation. J Biol Chem.

[28] Lelievre V (2001). Proliferative actions of natriuretic peptides on neuroblastoma cells. Involvement of guanylyl cyclase and non-guanylyl cyclase pathways. J Biol Chem.

[29] Li JK (2017). Long noncoding RNA MRCCAT1 promotes metastasis of clear cell renal cell carcinoma via inhibiting NPR3 and activating p38-MAPK signaling. Mol Cancer.

[30] Kent OA, Fox-Talbot K, Halushka MK (2013). RREB1 repressed miR-143/145 modulates KRAS signaling through downregulation of multiple targets. Oncogene.

[31] Janowski BA (2007). Activating gene expression in mammalian cells with promoter-targeted duplex RNAs. Nat Chem Biol.

[32] Place RF, Li LC, Pookot D, Noonan EJ, Dahiya R (2008). MicroRNA-373 induces expression of genes with complementary promoter sequences. Proc Natl Acad Sci USA.

