# Supplementary material for: MicroRNA-143 modulates the expression of Natriuretic Peptide Receptor 3 in cardiac cells
Source: Sci Rep. 2018 May 4;8:7055. doi: 10.1038/s41598-018-25489-3 (PMC5935707; doi:10.1038/s41598-018-25489-3)
Supplement: Supplementary file 1 — Supplementary Information [file 41598_2018_25489_MOESM1_ESM.docx]

**Supplementary Information**

**MicroRNA-143 modulates the expression of Natriuretic Peptide Receptor 3 in cardiac cells**

**Authors:** Juan Wang^1^, Kai Sing Tong^1^, Lee Lee Wong^1^, Oi-Wah Liew^1^, Divya Raghuram^1^, Arthur Mark Richards^1,2,3^, and Yei-Tsung Chen^1, *^

^1^ Cardiovascular Research Institute, Department of Medicine, Centre for Translational Medicine, Yong Loo Lin School of Medicine, National University of Singapore, Singapore, 117599, Singapore

^2^ Cardiac Department, National University Health System, Singapore, 119228, Singapore

^3^ Christchurch Heart Institute, University of Otago, Christchurch, 4345, New Zealand

^*^ Corresponding author: mdccyt@nus.edu.sg; yeitsung.chen@gmail.com

Supplemental data 1

Relative expression levels of miR-143 and miR-100 in different cardiovascular cells under normoxic and hypoxic environment with or without antagomiR-143 treatment. Cardiac cell line 2 and 3 were obtained from ATCC and PromoCell, respectively. The cell line derived from coronary artery smooth muscle was purchased from ATCC. Data are presented as mean ± SD, n = 3, Student’s t test, *p < 0.05.

Supplemental data 2

Relative expression levels of NPR3, NPPA, NPPC, and NR3C2 and CRHR2 in cardiac cell line 2 (ATCC) with miR-143 mimic or antagomiR-143 treatment. Data are presented as mean ± SD, n = 3, Student’s t test, *p < 0.05.

Supplemental data 3


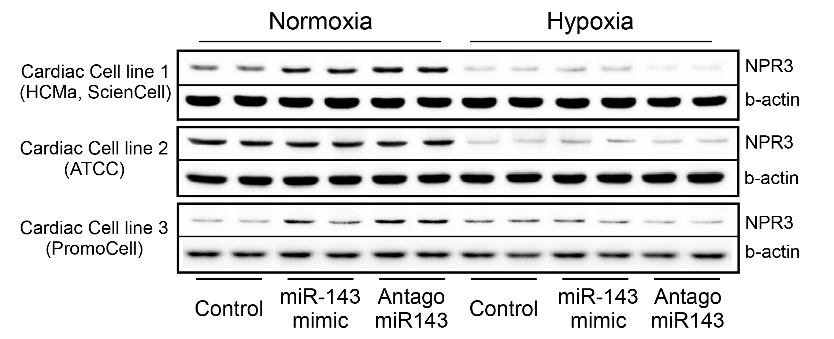


Western blot analysis of NPR3 protein levels in different cardiac cell lines under normoxic or hypoxic environments after overexpression or knockdown of endogenous miR-143 using miR-143 mimic or antagomir respectively.

Full images of unedited figures showing expression of HIF-1a and β actin in HCMa cells: supporting Figure 3b.

HIF-1a

1 2 3 4 5 6 7 8 9 10 11 12

β actin

1 2 3 4 5 6 7 8 9 10 11 12

Lane 1 and 2: Normoxia

Lane 3 and 4: Hypoxia (2 hours)

Lane 5 and 6: Hypoxia (4 hours)

Lane 7 and 8: Hypoxia (6 hours)

Lane 9 and 10: Hypoxia (8 hours)

Lane 11 and 12: Hypoxia (10 hours)

Full images of figures showing expression of β actin and NPR3 in different cardiac cell lines: supporting Figure S3.

HCMa





ATCC





Promocell





Lane 1 and 2: Normoxia control

Lane 3 and 4: Normoxia + miR-143 mimic

Lane 5 and 6: Normoxia + antago miR-143

Lane 7 and 8: Hypoxia control

Lane 9 and 10: Hypoxia + miR-143 mimic

Lane 11 and 12: Hypoxia + antago miR-143

Replicate images of unedited figures showing expression of β actin and NPR3 in different cardiac cell lines: supporting Figure S3.

HCMa 1 2 3 4 5 6 7 8 9 10 11 12

Beta-actin
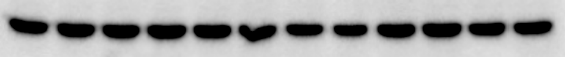


NPR3
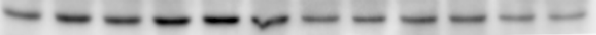


ATCC 1 2 3 4 5 6 7 8 9 10 11 12

Beta-actin
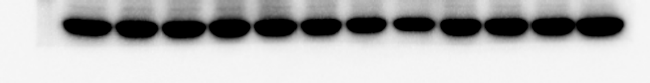


NPR3
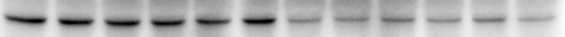


Promocell 1 2 3 4 5 6 7 8 9 10 11 12

Beta-actin
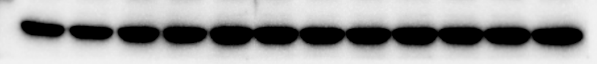


NPR3
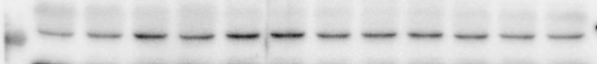


Lane 1 and 2: Normoxia control

Lane 3 and 4: Normoxia + miR-143 mimic

Lane 5 and 6: Normoxia + antago miR-143

Lane 7 and 8: Hypoxia control

Lane 9 and 10: Hypoxia + miR-143 mimic

Lane 11 and 12: Hypoxia + antago miR-143
